# Deep Learning Enabled Scoring of Pancreatic Neuroendocrine Tumors Based on Cancer Infiltration Patterns

**DOI:** 10.1007/s12022-025-09846-3

**Published:** 2025-01-23

**Authors:** Soner Koc, Ozgur Can Eren, Rohat Esmer, Fatma Ulkem Kasapoglu, Burcu Saka, Orhun Cig Taskin, Pelin Bagci, Nazmi Volkan Adsay, Cigdem Gunduz-Demir

**Affiliations:** 1 Department of Computer Engineering, Koc University, Istanbul, Turkey; 2 KUIS AI Center, Koc University, Istanbul, Turkey; 3 Department of Pathology, Koc University School of Medicine, Istanbul, Turkey; 4 Koc University Research Center for Translational Medicine, Istanbul, Turkey; 5 Department of Pathology, Marmara University Pendik Research and Training Hospital, Istanbul, Turkey; 6 Koc University School of Medicine, Istanbul, Turkey

**Keywords:** Pancreatic neuroendocrine tumors, Infiltration patterns, Graph neural networks, Deep learning, Artificial intelligence

## Abstract

Pancreatic neuroendocrine tumors (PanNETs) are a heterogeneous group of neoplasms that include tumors with different histomorphologic characteristics that can be correlated to sub-categories with different prognoses. In addition to the WHO grading scheme based on tumor proliferative activity, a new parameter based on the scoring of infiltration patterns at the interface of tumor and non-neoplastic parenchyma (*tumor-NNP interface*) has recently been proposed for PanNET categorization. Despite the known correlations, these categorizations can still be problematic due to the need for human judgment, which may involve intra- and inter-observer variability. Although there is a great need for automated systems working on quantitative metrics to reduce observer variability, there are no such systems for PanNET categorization. Addressing this gap, this study presents a computational pipeline that uses deep learning models to automatically categorize PanNETs for the first time. This pipeline proposes to quantitatively characterize PanNETs by constructing entity-graphs on the cells, and to learn the PanNET categorization using a graph neural network (GNN) trained on these graphs. Different than the previous studies, the proposed model integrates pathology domain knowledge into the GNN construction and training for the purpose of a deeper utilization of the tumor microenvironment and its architectural changes for PanNET categorization. We tested our model on 105 HE stained whole slide images of PanNET tissues. The experiments revealed that this domain knowledge integrated pipeline led to a 76.70% test set F1-score, resulting in significant improvements over its counterparts.

**Supplementary Information:**

The online version contains supplementary material available at 10.1007/s12022-025-09846-3.

## Introduction

Pancreatic neuroendocrine tumors (PanNET) are the second most common malignant neoplasms of the pancreas, and their incidence is on the rise. Compared to the more common adenocarcinomas of this organ, which are highly aggressive malignancies (currently the third leading cause of cancer-related deaths in the US [1]), PanNETs have a significantly better clinical outcome. However, their accurate management has been very problematic due to the highly variable behavior of the cases, with some patients cured with resection while others with similar disease succumb in a few years.

Currently, PanNETs without distant metastasis are treated with watchful waiting if < 2 cm, and pancreatectomy if > 2 cm as the standard treatment suggested by guidelines [2]. However, many patients on watchful waiting progress, and which ones will do so is a major concern. Similarly, predicting the behavior after resection, and determining which patients are more prone to progression and metastasis—and thus require further treatment—has been a major challenge. Conventional prognostic parameters applied for cancers in general, the grade and stage, have been of very limited value for resected PanNETs, with essentially all patients being placed into similar follow-up protocols without additional therapy. Whereas, while many of these patients with the same grade and stage experience rapid progression, until recently, there have not been any reliable parameters to further stratify this large group of patients who constitute about 90% of PanNETs [3, 4].

Recently, infiltration patterns at the interface between the tumor and the non-neoplastic parenchyma (*tumor-NNP interface*; a biologic phenomenon which has been used in other endocrine organs such as the thyroid or adrenal gland to define benign versus malignant categories) were tested in PanNETs [4]. It was found to be an independent prognostic factor even stronger than the grade. Subsequently, this was confirmed in other studies [3]. Of importance, this parameter is more readily applicable in routine diagnostic pathology, and unlike the grade evaluation, without necessitating cumbersome performance and assessment of special stains and substantial work by pathologists. It is something pathologists are accustomed to doing in other organs with relative ease. However, at the same time, as any other similar parameter, it is also vulnerable to subjectivity and quantitative illusions that can be corrected with digital methods. Automated systems based on quantitative metrics can further contribute to this end, decreasing subjectivity and yielding better reproducibility [5].

Deep learning has provided an effective tool to quantitatively analyze microscopic images of whole slides and correlate the characteristics of these images with the clinical behavior of the disease of interest [6, 7]. Typical steps followed in this analysis include dividing a whole slide image (WSI) into a grid of smaller patches, extracting features from these patches using a pretrained convolutional neural network (CNN), and classifying the WSI based on the extracted features. There are two main issues in this typical pipeline: First, the pixel-based nature of CNNs often overlooks histologically significant entities such as cells and glands, thereby limiting insight into the overall tissue microenvironment and consequently hindering the integration of established pathological knowledge at the entity-level into a neural network framework [8]. As a result, even though CNNs have shown promising results for simpler tasks in digital pathology such as differentiating tumorous, non-tumoral epithelial, and stromal regions, they may struggle with more challenging tasks such as tumor grading. To address this difficulty, it was proposed to represent tissue images as entity-graphs [9] and to use graph neural networks (GNNs) for feature extraction and tissue image classification [10]. The second issue concerns the processing of the patches (and their features) in a WSI to decide at the whole slide level, i.e., labeling the WSI with a diagnostic class using the patch features. In a supervised setting, the patch labels estimated by a classifier were usually aggregated to estimate the WSI class [11]. However, such aggregation can be problematic for WSI processing because a whole slide may contain heterogeneous regions; it is quite common to have non-neoplastic stromal and epithelial tissues within a whole slide showing a cancerous condition. Furthermore, the tumor may exhibit different characteristics in different cancerous regions, e.g., the characteristics in the center of the tumor mass and the tumor-NNP interface may be different, as in the case of PanNETs [4, 5]. In an unsupervised setting, the learning model was expected to select the most prominent patches to differentiate the WSIs of different diagnostic classes [12, 13]. Although this setting mitigates the problem of WSI heterogeneity to some extent, the previous studies did not use any sort of prior pathological knowledge in this selection but expected the model to learn this selection on its own. On the other hand, incorporating such knowledge into a supervised or unsupervised setting helps learn the WSI classification better, especially when the training data are limited.

This paper addresses these two issues for automatic infiltration pattern scoring of PanNETs, *for the first time in the literature*, by developing a new two-step pipeline. The first step uses a traditional pixel-based CNN to identify regions in a WSI that are most relevant for infiltration pattern scoring of PanNETs. The second step proposes conceptualizing these identified regions as entity graphs, where graph nodes represent cells in a region, and graph edges are assigned based on histomorphological similarities between the cells to emphasize their functional relations. This pipeline not only learns the PanNET infiltration pattern score with the GNN trained on these cell-graphs, but also proposes to incorporate the knowledge of the existence of different infiltration patterns observed at the tumor-NNP interface into the learning process by introducing a custom loss. This loss is designed to explicitly force the GNN to pay more attention to the cell-graphs closer to the tumor-NNP interface, which is parallel to the practice of a pathologist evaluating infiltration patterns.

## Materials and Methods

### Study Population and Data Preparation

**Fig. 1:**
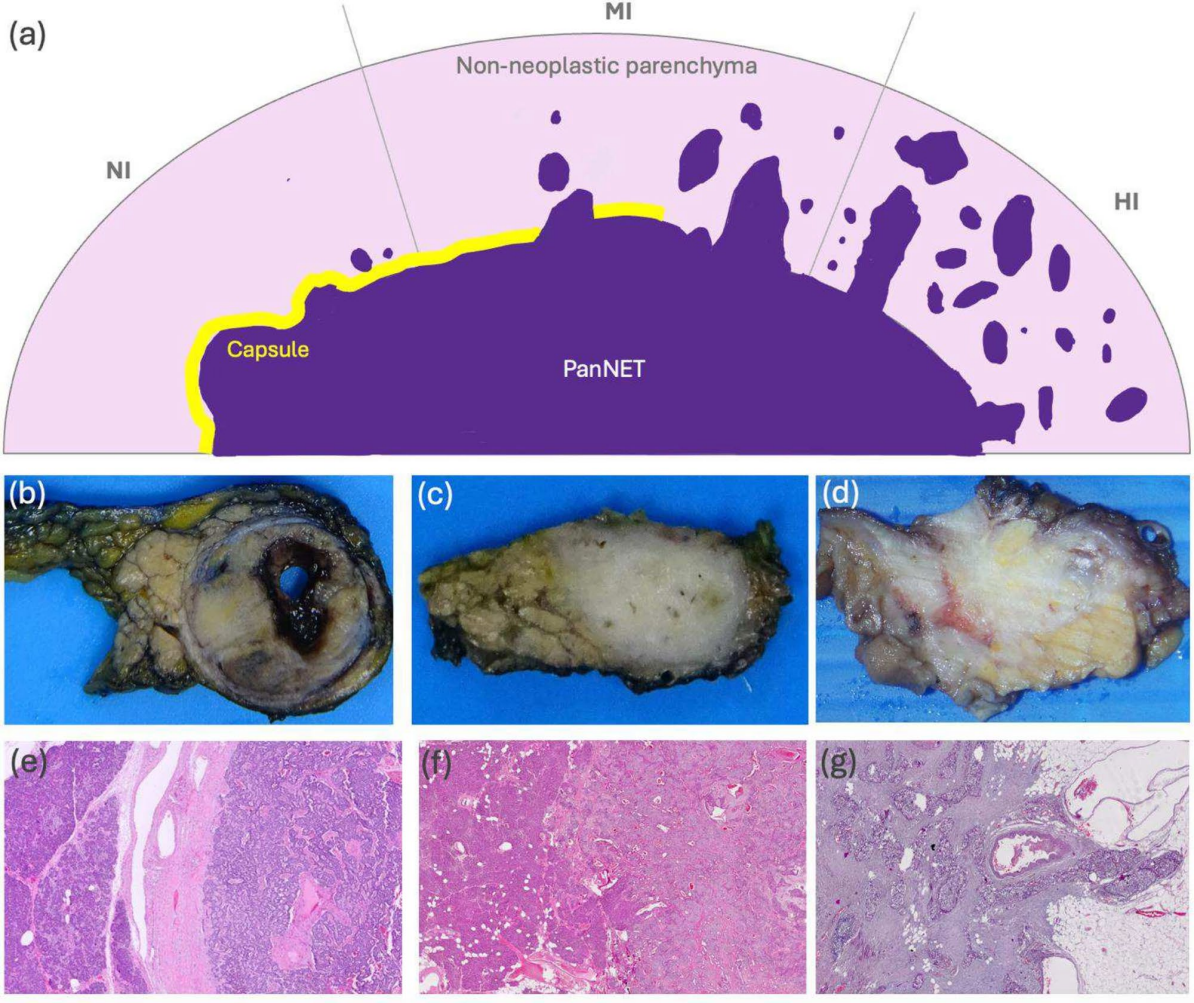
**a** Diagram of infiltration patterns NI (non/minimally infiltrative), MI (moderately infiltrative), and HI (highly infiltrative). **b**, **e** Macroscopic and microscopic examples of categories IPS1/NI; **c**, **f** IPS2/MI, and **d**, **g** IPS3/HI

The cohort used in this study (*n* = 35) was derived from a bigger cohort of surgical resections of PanNETs as explained previously [3] whose characteristics are summarized in Table 1. Briefly, the cohort consisting of 105 HE-stained WSIs was collected from 35 cases, three WSIs for each case, and digitized with a whole slide scanner at 0.25 µm/pixel resolution. Each case was annotated with an infiltration pattern score (IPS), from IPS1 to IPS3, as explained in the previous study [4]. In this scoring, three tumor-containing slides were selected for each case, representing the tumor sites with the lowest, intermediate, and highest degrees of infiltration. Then, each slide was subjected to histopathological examination and individually scored from 1 to 5 according to the infiltration patterns observed at its tumor-NNP interface. Afterward, the case was assigned an infiltration pattern score based on the total score of the three slides: The case was labeled as “non/minimally infiltrative (IPS1)” if the total score was from 3 to 6, “moderately infiltrative (IPS2)” if it was from 7 to 9, and “highly infiltrative (IPS3)” if it was from 10 to 15. A schematic representation of infiltration patterns “NI” (non/minimally infiltrative), “MI” (moderately infiltrative), and “HI” (highly infiltrative), as well as macroscopic and microscopic correlates, are illustrated in Fig. 1.

**Table 1:** Clinicopathological characteristics of the PanNET cohort used in this study. Note that infiltration pattern scores were assigned by following the recently proposed scoring methodology [4]

Study characteristics	Patient (*n* = 35)
Tumor size	
Median size (range), cm	3.75 (1.0–11.0)
Size < 2*.*0, *n* (%)	7 (20.0%)
Size ≥ 2*.*0, *n* (%)	23 (65.7%)
No data, *n* (%)	5 (14.3%)
Age	
Median age (range), years	55 (17.0–81.0)
Age < 50, *n* (%)	11 (31.4%)
Age ≥ 50, *n* (%)	22 (62.9%)
No data, *n* (%)	2 (5.7%)
Gender	
Male, *n* (%)	14 (40.0%)
Female, *n* (%)	18 (51.4%)
No data, *n* (%)	3 (8.6%)
Recurrence	
Recurrence-free, *n* (%)	29 (82.9%)
Recurred, *n* (%)	4 (11.4%)
No data, *n* (%)	2 (5.7%)
Infiltration pattern score	
IPS1, *n* (%)	7 (20.0%)
IPS2, *n* (%)	18 (51.4%)
IPS3, *n* (%)	10 (28.6%)
Lymphovascular invasion	
Present, *n* (%)	15 (42.9%)
Absent, *n* (%)	18 (51.4%)
No data, *n* (%)	2 (5.7%)
Perineural invasion	
Present, *n* (%)	13 (37.1%)
Absent, *n* (%)	20 (57.1%)
No data, *n* (%)	2 (5.7%)
Lymph node metastasis	
Present, *n* (%)	14 (40.0%)
Absent, *n* (%)	14 (40.0%)
No data, *n* (%)	7 (20.0%)
Distant metastasis	
Present, *n* (%)	9 (25.7%)
Absent, *n* (%)	19 (54.3%)
No data, *n* (%)	7 (20.0%)

In the proposed pipeline, the first step trains a CNN classifier to select representative tumor-NNP interface patches from a WSI, and the second step trains a GNN classifier to categorize these selected patches. The case-level infiltration pattern score (IPS1, IPS2, or IPS3) was used as the ground truth label for all representative patches of this case to train the GNN. Additionally, a small subset of WSIs were patch-level annotated for training the CNN classifier at the first step (see Supplementary Figure S1).

### Model Development

**Fig. 2:**
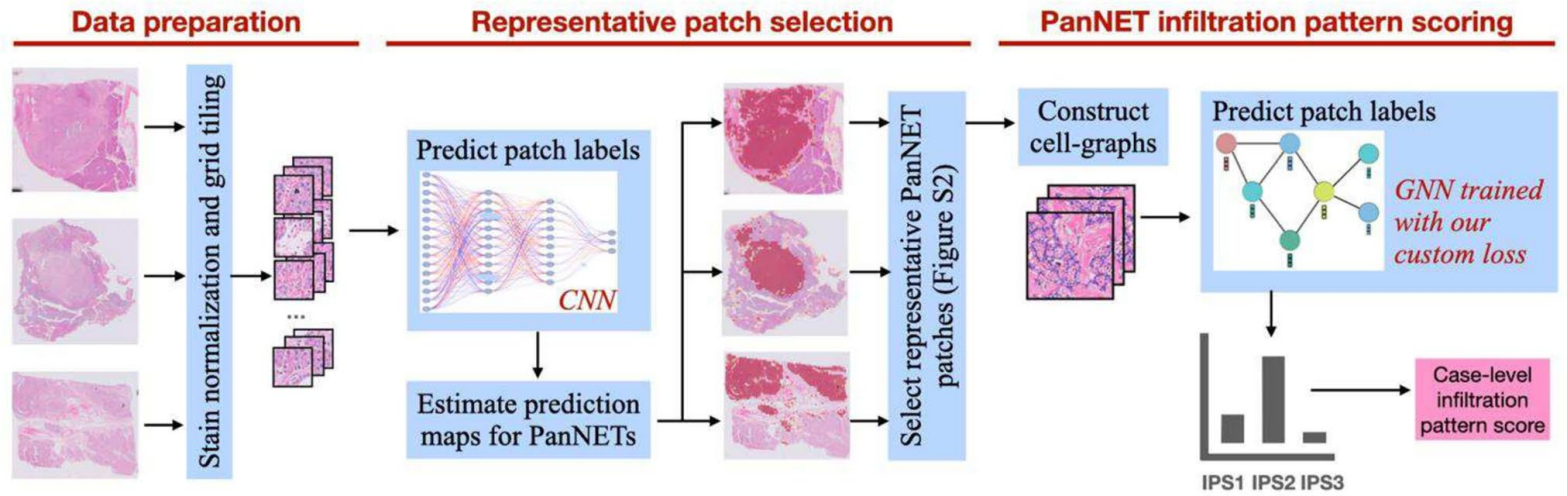
Overview of the proposed infiltration pattern scoring pipeline. After extracting image patches from the three stain-normalized WSIs of a given case, they are fed to the CNN classifier to select representative tumor-NNP interface patches from the WSIs. Then, each patch is modeled by constructing a cell-graph on its nuclei, and its infiltration pattern score was estimated by the GNN classifier. At the end, estimated scores of all selected patches from the three WSIs are voted to predict the case-level infiltration pattern score

The proposed pipeline consists of two steps: selection of representative tumor-NNP interface patches from a WSI and infiltration pattern scoring of the WSI based on the selected patches. The schematic overview of this pipeline is shown in Fig. 2 and the details are provided in the following subsections.

#### Representative Patch Selection

In this step, a CNN classifier is trained to distinguish PanNET regions from the others. Later, for any WSI, this trained classifier is used to identify its PanNET regions, and a subset of these regions is selected as the representative tumor-NNP interface. The patches belonging to this interface are then used for PanNET categorization in the next step (see Supplementary Sect. 1.2.1 for extended details).

**Fig. 3:**
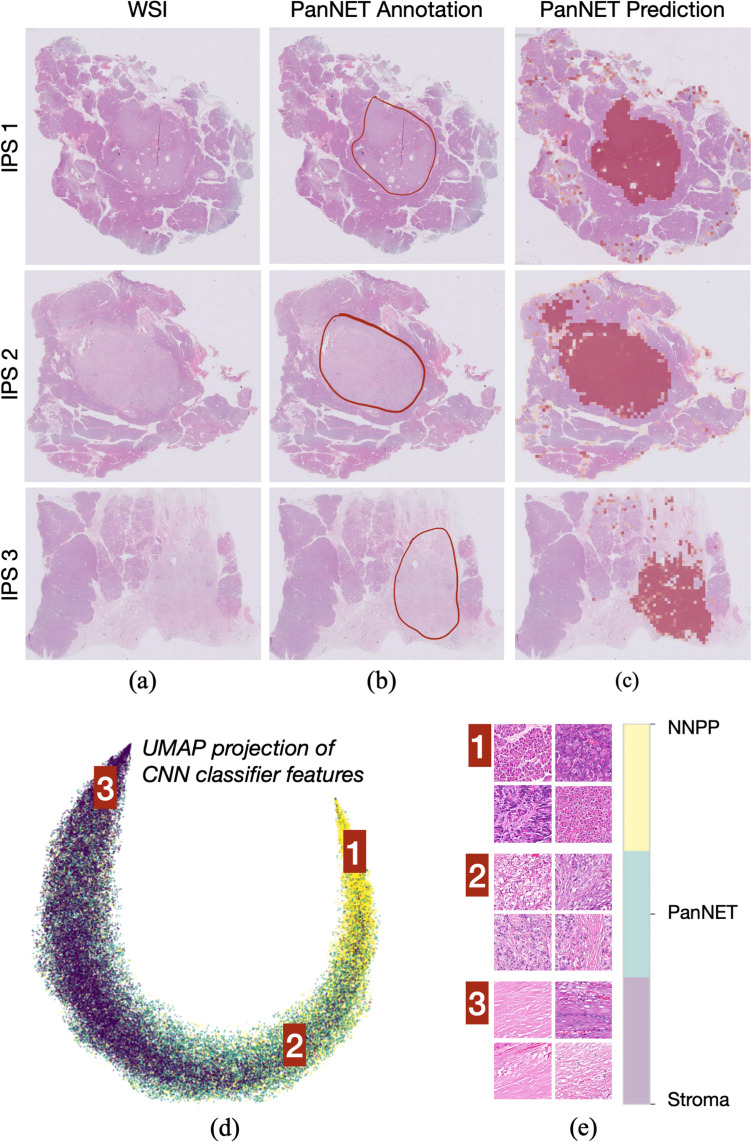
Visual results on three example WSIs. **a** Original WSIs with IPS1, IPS2, and IPS3 categorization, respectively. **b** Pathologist annotations for the largest PanNET region in each WSI. **c** PanNET regions predicted by the trained CNN classifier in the first step. **d** UMAP projection of the features used by the CNN classifier after training it to differentiate between three classes of stroma, NNPP, and PanNET. **e** Example patches selected from the focus of the CNN tissue classifier

The trained CNN classifier is used to predict the classes of patches in each WSI and to generate a PanNET prediction map to identify the largest tumor region of interest (TRoI) in the WSI (Fig. 3). Afterward, the patch selector identifies PanNET patches in the largest TRoI that are most likely to belong to the tumor-NNP interface. To do this, it first takes all PanNET patches on the border of the largest TRoI, i.e., if the patch belongs to the largest TRoI and is adjacent to another patch that the CNN classifier labeled with the NNPP or stroma class. To enrich the training dataset with more samples from the tumor-NNP interface, this step extends the patch selection up to maximum distance hops towards the tumor center in the prediction map (see Supplementary Figure S2). This subset of patches, together with their hop distances (used to weight their contributions in the deep learning model) serves as the input for the next step, infiltration pattern scoring.

#### Infiltration Pattern Scoring of PanNETs

The proposed pipeline relies on modeling infiltration patterns at the tumor-NNP interface by making use of a cell-graph representation and learning these patterns through a graph neural network (GNN) that is optimized with our proposed pathology domain knowledge integrated loss function, which we call *InfiltrationLoss* (see Supplementary Sect. 1.2.2).

A cell-graph is intended to represent the microenvironment of cells, where graph nodes symbolize the cell nuclei, capturing their morphological features, and graph edges represent cellular interactions, reflecting both histomorphological and spatial relationships between the cells. Its construction involves three main steps: nucleus detection for node identification, feature extraction for node (cell nuclei) embeddings, and topology representation for edge assignment among cells. In this work, cell locations are detected using Hover-Net, a nucleus segmentation network [14, 15]. The centroids of the nuclei detected by the Hover-Net model are used as the coordinates of the nodes in the cell-graph.

The selected PanNET patches are then classified into the three infiltration pattern scores (namely, IPS1, IPS2, and IPS3) by making an inference on their corresponding cell-graphs using a GNN classifier. Then, the GNN is trained to minimize the proposed domain knowledge integrated loss function, *InfiltrationLoss*. See Supplementary Figure S4 for further graph-based classifier details and analysis of the effectiveness of these selections on the performance. Note that the infiltration pattern scoring methodology [4] uses three WSIs to characterize a case. Thus, the representative PanNET patches selected for all three WSIs are put into the same bag, the grades of these patches are estimated by the trained GNN, and then they are voted over the bag to predict the case-level infiltration pattern score.

The proposed *InfiltrationLoss* prioritizes learning the patterns of the training patches as they get closer to the tumor-NNP interface. Thus, it introduces a distance-based regularization term in the loss function. This term gives the highest importance to the border patches and exponentially decreases that importance as patches move away from the tumor-NNP interface. The exponential decrease provides a better focus on smaller distances, which correspond to the patches closer to the tumor-NNP interface (see Supplementary Figure S3). Training the GNN to optimize this modified loss over all training patches enhances the training focus on the edges of the tumor-NNP interface, which is consistent to the practice of pathologists for infiltration pattern scoring of PanNETs [4]. Extended details about the graph construction and proposed *InfiltrationLoss* are provided in Supplementary Sect. 1.2.2.

#### Evaluation Metrics

For the quantitative evaluation of the classification tasks in our pipeline, we employed the weighted F1-score metric, which balances precision and recall. True positive (TP), false positive (FP), and false negative (FN) instances were identified at the appropriate level, either patch-level for tissue classification or case-level for PanNET classification. Precision was calculated as TP/ (TP + FP), recall as TP/(TP + FN), and F1-score as (2 × precision × recall)/(precision + recall). In order to account for class imbalance often encountered in histopathological datasets, we computed the weighted F1-score by averaging the F1-scores of individual classes weighted by their frequency in the dataset.

## Results

This section provides a quantitative evaluation of the two classification tasks in our pipeline: tissue-level classification of patches and case-level classification of PanNETs. For the first task, the CNN classifier was used to classify patches into three classes: PanNET, NNPP, and stroma. It was trained and validated on 18 K image patches selected from 20 annotated WSIs (see Supplementary Figure S1). Our experiments led to 98.30, 98.49, and 89.23 percent average F1-scores on test instances for the PanNET, NNPP, and stroma classes, respectively; the weighted F1-score was 95.52. These scores showed that the CNN classifier was successful in accurately identifying the majority of PanNET patches. Here, it is worth noting that this classification was used to select representative patches for the next step but was not directly used for infiltration pattern scoring of PanNETs. Thus, correctly locating most of PanNET patches can be sufficient, and mispredictions can be tolerated to a certain extent for the final PanNET categorization. This can also be seen visually in Figs. 3a–c, in the prediction maps generated for three example WSIs. They show that the features learned by the CNN provided sufficient information for accurate patch classification. We also visualized the distributions of these features for the three classes to understand their effectiveness. For that, we reduced the dimension of the features to two using the technique called uniform manifold approximation and projection (UMAP). These projections and example patches selected from the focus of the CNN classifier are shown in Figs. 3d–e, respectively.

After estimating PanNET patches using the CNN classifier, those close to the tumor-NNP interface were selected as representative patches, as this region plays a critical role in determining tumor behavior. These representative patches were further classified into one of the three classes: IPS1, IPS2, and IPS3. Then, the infiltration pattern score of a case was predicted by voting on the estimated classes of the representative patches selected from the three WSIs of this case. Since the number of cases (patients) was relatively small, to ensure the reliability of our results and avoid depending too heavily on any single group of samples, we repeatedly tested our approach on different subsets of the data (five-fold stratified cross-validation). The average weighted F1-score was 76.70 percent (see Supplementary Results for further details).

## Discussion

The literature includes deep learning-based studies for the characterization and classification of pancreatic tissues. They mostly focused on the differentiation of pancreatic ductal adenocarcinoma (PDAC) from healthy pancreas and other pathologies. In these studies, CNNs were commonly used for patch-level classification [16–18]. Then, the outputs of these classifiers were used to localize areas with a high tumor content [16, 17] or accumulated for PDAC classification at the whole slide level. CNN classifiers were also used to extract features from tissue patches, which were later employed by rule-based methods [18] and in a more holistic interpretation considering an entire WSI [19]. It was also proposed to construct a graph on patches to capture the complex relationships among different areas of a WSI, and the extracted features were used to quantify the patches in this graph [20]. These studies led to promising results for PDAC differentiation. On the other hand, there exists only one deep learning-based study reported for PanNET characterization. This study aimed to predict metastatic risk using two successive CNNs. The first CNN was trained to identify PanNET patches across a WSI, then the other one was trained to estimate the metastatic risk at the WSI [21]. Note that there was another study on neuroendocrine tumors; however, this study was not to characterize PanNETs, but to predict the site of origin for well-differentiated neuroendocrine tumors using a CNN classifier as an alternative to using immunohistochemistry [22]. As opposed to these previous studies, this paper proposed a computational pipeline for the automated categorization of PanNETs, for the first time. It relied on modeling the following knowledge about PanNETs: different infiltration patterns are observed at the interface of tumor and non-neoplastic parenchyma (*tumor-NNP interface*), and this can be used as an alternative histomorphologic parameter for PanNET categorization [4, 5]. To model this pathological knowledge and integrate it into a learning model, it proposed to select representative patches from the tumor-NNP interface, represent these patches as cell-graphs, and introduce the *InfiltrationLoss* function to force a GNN classifier to pay increasing attention to the patches (cell-graphs) closer to the tumor-NNP interface.

Our experiments revealed that the cell-graph representation was more successful than the direct use of patch pixels for PanNET infiltration pattern scoring. One explanation for this difficulty lies in the challenge for AI-based models to balance the use of fine detail with the broader tissue context in HE stained tissue sections. Focusing too closely (at a very high magnification) helps visualize individual cellular features but limits the view of the surrounding tissue architecture. On the contrary, stepping back to a lower magnification provides an overall impression of the tissue’s structure, however, makes it harder to appreciate subtle cellular details [23]. Although state-of-the-art AI-based methods can use both local details and contextual cues [24], they may still find it challenging to perform more nuanced tasks, such as tumor grading, which demand a deeper understanding of the tissue microenvironment as a whole. We also observed this phenomenon in our experiments. For comparison, we employed two commonly used CNN-based settings, different than ours, for WSI classification. In the first one, the classifier relied on pathologist-labeled examples for guidance (like a teaching process, which is called supervised learning). In the second one, the classifier attempted to identify patterns on its own without relying on these labels (which is called unsupervised learning). Table 2 shows the comparative results between our proposed pipeline (*InfiltrationLoss-*GNN) and these two settings, namely Patch-CNN and CLAM [12], respectively. Graph neural networks (GNNs) have been proposed to mitigate the shortcomings of CNNs. Earlier studies trained their GNNs on the entity-graphs constructed on cells, which were initially characterized with either human-defined features [25] or those identified by deep learning methods [25–27]. Recent work has extended the use of graph-based methods to represent not only individual cells, but also larger tissue patches [28, 29], entire tissue regions [30, 31], and even complex structures that combined both tissue and cellular information [30]. These advancements provided a more complete view of the tissue architecture, capturing details at multiple levels. In Table 2, we also compared our proposed GNN-based pipeline with other graph-based models [25, 29, 30]. This table reveals that our proposed model resulted in a significantly higher weighted F1-score compared to all these graph-based methods, which demonstrated the power of incorporating pathology domain knowledge into the design of a learning model. Please see Supplementary Results for further details of the comparison algorithms and the results.

**Table 2:** F1-scores of our proposed *InfiltrationLoss*-GNN and the comparison algorithms. These were the average scores obtained over the 15 runs (five folds and three runs for each fold) and their standard deviations

	IPS1	IPS2	IPS3	Weighted-F1
*InfiltrationLoss*-GNN	61.54 ± 0.00	84.97 ± 1.31	72.46 ± 2.13	76.70 ± 0.07
Patch-CNN	40.12 ± 2.68	72.95 ± 2.32	51.94 ± 4.01	60.38 ± 0.37
CLAM [12]	50.13 ± 3.14	76.61 ± 2.02	54.58 ± 1.69	65.02 ± 1.33
Patch-GCN [29]	52.22 ± 1.93	77.82 ± 2.16	53.70 ± 3.21	65.81 ± 0.32
CGC-Net [25]	57.14 ± 5.44	78.94 ± 2.07	55.55 ± 1.65	67.90 ± 0.45
HACT-Net [30]	57.34 ± 4.11	85.01 ± 2.56	65.50 ± 2.03	73.90 ± 0.32

**Fig. 4:**
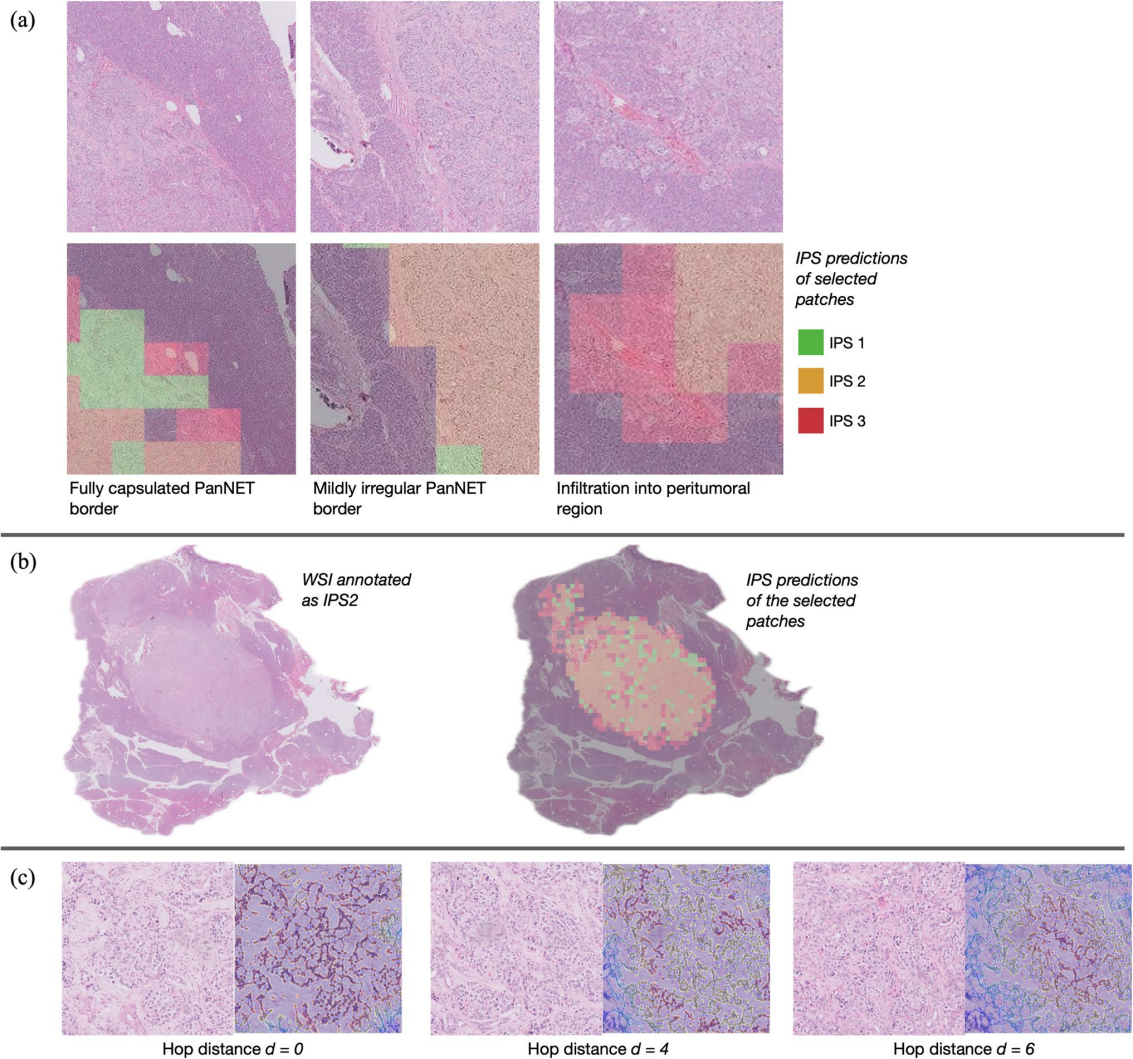
**a** IPS predictions of the GNN classifier for patches at the tumor-NNP interface. These regions are taken from the PanNET borders exhibiting different infiltration characteristics. Note that these are not the pathologist-annotated PanNET patches, but those automatically annotated with the PanNET class by the first step CNN classifier. Thus, it may contain some incorrectly selected patches. **b** For an example WSI, the prediction map containing IPS predictions of patches. Likewise, these patches are those automatically annotated with the PanNET class by the first step CNN classifier. **c** Representative PanNET patches selected closer to (hop distance *d* = 0) and farther away from (*d* = 4 and *d* = 6) the tumor-NNP interface and the attention heatmaps calculated on their nodes by the Graph-GradCAM [32] visualization technique. The redder a node is, the more attention it gets from the GNN classifier

In the experiments, we observed that our proposed pipeline could produce different infiltration pattern predictions for the PanNET borders exhibiting different characteristics (Fig. 4a). This was more effective to better estimate the IPS classes of patches in each WSI, which in turn led to more accurate case-level IPS categorizations (Fig. 4b). This improvement was attributed to representing patches with cell-based graphs, employing a GNN classifier to analyze these graphs, and training the GNN using a loss function informed by pathology domain knowledge. This guided the model to pay more attention to patches (cell-graphs) near the tumor–NNP interface. To further understand the model’s decision-making process, we utilized a visualization technique [32] to produce an attention heatmap, highlighting the nodes the trained GNN classifier focuses on when determining a class. Figure 4c shows the attention heatmaps of some patches generated for our trained GNN classifier (in a heatmap, the redder a node is, the more attention it gets from the GNN classifier). In this figure, we specifically selected representative PanNET patches closer to and farther away from the tumor-NNP interface to compare the attention paid by the classifier. This figure demonstrates that the proposed GNN classifier paid increasingly more attention to a higher number of nodes (there were more reddish but less blueish nodes) in cell-graphs constructed for patches when one moved closer to the tumor-NNP interface.

This work proposed an effective tool for the categorization of infiltration patterns observed at the interface of tumor and non-neoplastic parenchyma. Its experimental validation was limited for PanNETs and for the cohort selected from our previous study [4]. As one research direction of this study, the generalizability of the proposed model could be tested for other tumor types for which such infiltration patterns may be important, as well as for the other cohorts. Another limitation of this study is that it relies on pathologist-provided training samples in the early stages of the proposed computational pipeline. Although it was enough to use rough annotations on only a small number of WSIs, this required annotation could be completely excluded from the pipeline and the PanNET regions could be identified in an unsupervised setting. This might be another future research direction. Moreover, this study used established methods to represent the interactions between cells and their surroundings. However, exploring more flexible ways to capture the complexity of the tissue environment may yield richer insights. By refining how we model these relationships, we might gain a more comprehensive understanding of infiltration patterns and their implications in clinical practice. For an extended discussion on this issue, please refer to the Supplementary Discussion.

Lastly, it should be reiterated here that while it is currently not recognized and incorporated into management algorithms, infiltration pattern (IP) grading at the tumor-host interface has been shown to be an independent prognosticator in our prior study [4]. This was later amply supported in other studies as well [5]. Considering that in other endocrine organs, circumscription is the main determinant of whether a tumor is benign vs malignant, it is natural that this parameter will be an important part of PanNET management. This is not only applicable for resected PanNETs, but potentially in pre-operative diagnosis and management of PanNETs as IP grading gets adopted by radiologists and EUS endoscopists. Currently, virtually all non-metastatic PanNETs are placed into the same imprecise management protocols, there is a great need for proper triaging of PanNETs into more effective and accurate management algorithms and IP grading supported by AI offers an invaluable opportunity in this regard. Especially for the PanNETs that are < 2 cm, which are now worldwide placed into watchful waiting protocols, this parameter could prove of vital value considering that a subset of those patients develop metastasis during the observation period., Needless to say that AI support for this parameter, which has both subjective quantitative and qualitative, and which is not something routinely performed by pathologists, could be extremely valuable in the application of this grading system.

This pilot study both documented the strong correlation of AI-conducted IP grading with histopathologic evaluation and established the applicability of this approach, in addition to highlighting the improvements needed for it to be incorporated into daily practice. Naturally, the findings of this proof-of-concept study will have to be confirmed in a larger series of cases. More importantly, future studies are warranted to focus on evaluating this prognostic and predictive value in comparison with other clinically relevant parameters.

## Supplementary Information

Below is the link to the electronic supplementary material.

Supplementary file1 (PDF 9885 KB)

## Data Availability

No datasets were generated or analysed during the current study.

## Data Availability

No datasets were generated or analyzed during the current study.

## References

[1] Siegel RL, Miller KD, Wagle NS, Jemal A (2024) Cancer statistics, 2024. CA Cancer J Clin. 10.3322/caac.21763 36633525

[2] Kos-Kudła B, et al. (2023) European Neuroendocrine Tumour Society (ENETS) 2023 guidance paper for nonfunctioning pancreatic neuroendocrine tumours. J Neuroendocrinol 35(12):e13343. 10.1111/jne.13343 37877341 10.1111/jne.13343

[3] Eren OC, et al. (2024) Subgrading of G2 pancreatic neuroendocrine tumors as 2A (Ki67 3% to <10%) versus 2B (10% to ≤20%) identifies behaviorally distinct subsets in keeping with the evolving management protocols. Ann Surg Oncol 1–11. 10.1245/s10434-024-13049-x 10.1245/s10434-024-15632-y PMC11413052 38955993

[4] Taskin OC, et al. (2021) Infiltration pattern predicts metastasis and progression better than T-stage and grade in pancreatic neuroendocrine tumors: a proposal for a novel infiltration-based morphologic grading. Mod Pathol 1–9. 10.1038/s41379-021-00838-8 10.1038/s41379-021-00995-4 34969955

[5] Schiavo Lena M, et al. (2023) Infiltrative growth predicts the risk of recurrence after surgery in well-differentiated non-functioning pancreatic neuroendocrine tumors. Endocr Pathol 34:142–155. 10.1007/s12022-023-09978-2 36564582 10.1007/s12022-022-09745-x

[6] Fu Y, et al. (2019) Pan-cancer computational histopathology reveals mutations, tumor composition and prognosis. Nat Cancer 1:800–810. 10.1038/s41571-019-0304-8 10.1038/s43018-020-0085-8 35122049

[7] Litjens G, et al. (2016) Deep learning as a tool for increased accuracy and efficiency of histopathological diagnosis. Sci Rep 6:26286. 10.1038/srep26286 27212078 10.1038/srep26286 PMC4876324

[8] Hagele M, et al. (2020) Resolving challenges in deep learning-based analyses of histopathological images using explanation methods. Sci Rep 10:6423. 10.1038/s41598-020-63371-7 32286358 10.1038/s41598-020-62724-2 PMC7156509

[9] Gunduz C, Yener B, Gultekin SH (2004) The cell graphs of cancer. Bioinformatics 20:i145–i151. 10.1093/bioinformatics/bth921 15262793 10.1093/bioinformatics/bth933

[10] Anand D, Gadiya S, Sethi A (2020) Histographs: Graphs in histopathology. SPIE Med Imaging Digit Pathol 11320:150–155. 10.1117/12.2548741

[11] Mercan C, et al. (2019) From patch-level to ROI-level deep feature representations for breast histopathology classification. SPIE Med Imaging Digit Pathol 10956:86–93. 10.1117/12.2511998

[12] Lu MY, et al. (2021) Data-efficient and weakly supervised computational pathology on whole-slide images. Nat Biomed Eng 5:555–570. 10.1038/s41551-020-00682-7 33649564 10.1038/s41551-020-00682-w PMC8711640

[13] Shao Z, et al. (2021) TransMIL: Transformer based correlated multiple instance learning for whole slide image classification. Adv Neural Inf Process Syst 34:2136–2147.

[14] Graham S, et al. (2019) Hover-Net: Simultaneous segmentation and classification of nuclei in multitissue histology images. Med Image Anal 58:101563. 10.1016/j.media.2019.101563 31561183 10.1016/j.media.2019.101563

[15] Kumar N, et al. (2017) A dataset and a technique for generalized nuclear segmentation for computational pathology. IEEE Trans Med Imaging 36:1550–1560. 10.1109/TMI.2017.2677499 28287963 10.1109/TMI.2017.2677499

[16] Kronberg RM, et al. (2022) Communicator-driven data preprocessing improves deep transfer learning of histopathological prediction of pancreatic ductal adenocarcinoma. Cancers 14:1964. 10.3390/cancers14081964 35454869 10.3390/cancers14081964 PMC9031738

[17] Kriegsmann M, et al. (2021) Deep learning in pancreatic tissue: Identification of anatomical structures, pancreatic intraepithelial neoplasia, and ductal adenocarcinoma. Int J Mol Sci 22. 10.3390/ijms222111424 10.3390/ijms22105385 PMC8160892 34065423

[18] Fu H, et al. (2021) Automatic pancreatic ductal adenocarcinoma detection in whole slide images using deep convolutional neural networks. Front Oncol 11:665929. 10.3389/fonc.2021.665929 34249702 10.3389/fonc.2021.665929 PMC8267174

[19] Carrillo-Perez F, et al. (2023) Performance comparison between multi-center histopathology datasets of a weakly-supervised deep learning model for pancreatic ductal adenocarcinoma detection. Cancer Imaging 23:66. 10.1186/s40644-023-00485-w 37365659 10.1186/s40644-023-00586-3 PMC10294485

[20] Li B, et al. (2022) Differentiation of pancreatic ductal adenocarcinoma and chronic pancreatitis using graph neural networks on histopathology and collagen fiber features. J Pathol Inform 13:100158. 10.4103/jpi.jpi_45_21 36605110 10.1016/j.jpi.2022.100158 PMC9808020

[21] Klimov S, et al. (2021) Predicting metastasis risk in pancreatic neuroendocrine tumors using deep learning image analysis. Front Oncol 10:593211. 10.3389/fonc.2020.593211 33718106 10.3389/fonc.2020.593211 PMC7946991

[22] Redemann J, et al. (2020) Comparing deep learning and immunohistochemistry in determining the site of origin for well-differentiated neuroendocrine tumors. J Pathol Inform 11:32. 10.4103/jpi.jpi_16_20 33343993 10.4103/jpi.jpi_37_20 PMC7737494

[23] Sirinukunwattana K, et al. (2018) Improving whole slide segmentation through visual context - a systematic study. Med Image Comput Comput Assist Interv 192–200. 10.1007/978-3-030-00928-1_22

[24] Tellez D, et al. (2019) Quantifying the effects of data augmentation and stain color normalization in convolutional neural networks for computational pathology. Med Image Anal 58:101544. 10.1016/j.media.2019.101544 31466046 10.1016/j.media.2019.101544

[25] Zhou Y, et al. (2019) CGC-Net: Cell graph convolutional network for grading of colorectal cancer histology images. IEEE/CVF Int Conf Comput Vis Work.

[26] Jaume G, et al. (2021) Quantifying explainers of graph neural networks in computational pathology. Proc IEEE/CVF Conf Comput Vis Pattern Recognit 8106–8116.

[27] Chen RJ, et al. (2020) Pathomic fusion: An integrated framework for fusing histopathology and genomic features for cancer diagnosis and prognosis. IEEE Trans Med Imaging 41:757–770. 10.1109/TMI.2020.2968528 10.1109/TMI.2020.3021387 PMC10339462 32881682

[28] Aygunes B, et al. (2020) Graph convolutional networks for region of interest classification in breast histopathology. SPIE Med Imaging Digit Pathol 11320:134–141. 10.1117/12.2548776

[29] Chen RJ, et al. (2021) Whole slide images are 2D point clouds: Context-aware survival prediction using patch-based graph convolutional networks. Med Image Comput Comput Assist Interv 339–349.

[30] Pati P, et al. (2022) Hierarchical graph representations in digital pathology. Med Image Anal 75:102264. 10.1016/j.media.2021.102264 34781160 10.1016/j.media.2021.102264

[31] Anklin V, et al. (2021) Learning whole-slide segmentation from inexact and incomplete labels using tissue graphs. Med Image Comput Comput Assist Interv 636–646.

[32] Jaume G, et al. (2021) HistoCartography: A toolkit for graph analytics in digital pathology. MICCAI Workshop Comput Pathol 117–128.

