# Supplementary material for: Deep Learning Enabled Scoring of Pancreatic Neuroendocrine Tumors Based on Cancer Infiltration Patterns
Source: Endocr Pathol. 2025 Jan 23;36(1):2. doi: 10.1007/s12022-025-09846-3 (PMC11757657; doi:10.1007/s12022-025-09846-3)
Supplement: Supplementary file 1 — Supplementary file1 (PDF 9885 KB) [file 12022_2025_9846_MOESM1_ESM.pdf]

Supplementary Information

Deep Learning-Enabled Scoring of Pancreatic Neuroendocrine Tumors Based on Cancer Infiltration Patterns

Soner Koc[1,2,*], Ozgur Can Eren[3,4,*], Rohat Esmer[3,4], Fatma Ulkem Kasapoglu[1,2], Burcu Saka[3,4]
Orhun Cig Taskin[3,4], Pelin Bagci[5]
Nazmi Volkan Adsay[3,4,6] and Cigdem Gunduz-Demir[1,2,6]
[1]Department of Computer Engineering and KUIS AI Center, Koç University
[2]KUIS AI Center, Koç University
[3]Department of Pathology, Koç University School of Medicine and Koç University Research Center for Translational Medicine (KUTTAM)
[4]Koc University Research Center for Translational Medicine, Istanbul, Turkey
[5]Department of Pathology, Marmara University Pendik Research and Training Hospital, Istanbul, Turkey
[6]Koç University School of Medicine
[*]Contributed equally

‡

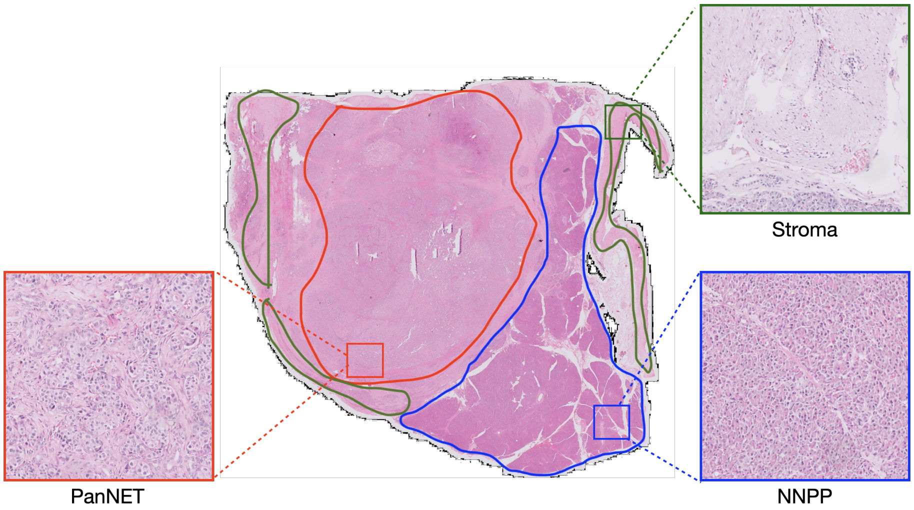

Figure S1: Illustration of the annotation process for training the CNN classifier at the first step. Three example microscopic images of tissue samples labeled with the PanNET, non-neoplastic pancreas parenchyma (NNPP), and stroma class are also given.

1 Supplementary Materials and Methods

1.1 Study population and data preparation

Twenty WSIs were patch-level annotated for training the CNN classifier, as seen in Figure S1, using the QuPath tool[1]. Three class labels were used for this annotation: PanNET, non-neoplastic pancreas parenchyma (NNPP), and stroma. Then, from the annotated regions, 1024×1024 patches were gathered such that the number of patches selected for each class is similar.

Images of hematoxylin-eosin (HE) stained tissues inherently exhibit variability in appearance due to factors such as diverse specimen preparation methods, staining techniques, fixation traits, and imaging device characteristics that adversely affect subsequent computational techniques[11, 13]. To reduce this variability and have more standardized inputs to the networks, all images were normalized using an unsupervised, reference-free stain normalization algorithm. This algorithm was previously developed to normalize histology slides[9] and was easy to use since it did not require model training and was computationally inexpensive.

1.2 Model development

1.2.1 Representative patch selection

The patches for the CNN classifier are prepared as follows: background regions are removed from a stained-normalized WSI using the Otsu's thresholding technique, and the remaining parts are divided into a grid of non-overlapping patches with a pixel resolution of 1024×1024. The CNN

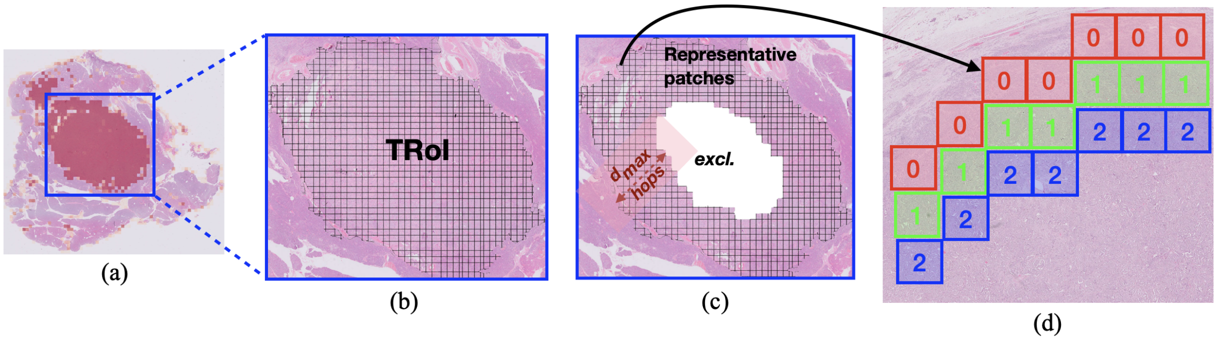

Figure S2: Illustration of the representative patch selection. (a) PanNET prediction map produced by the CNN classifier. (b) Largest tumor region of interest (TRoI). (c) Representative PanNET patches were selected close to the border of the largest TRoI. (d) It is an illustration of selecting the border patches (red) and extending them up to one and two hops (green and blue). For these patches, hop distances d are 0, 1, and 2, respectively.

classifier is then designed to assign patches to one of the three categories: PanNET, non-neoplastic pancreas parenchyma (NNPP), and stroma. In this design, transfer learning is used by employing the ResNet-18 model pre-trained on the ImageNet dataset. In particular, the encoder of this model together with its weights are directly taken, and a multilayer perceptron (MLP) with two layers, each containing 128 hidden units, is put at the end of this encoder. Then, all weights are finetuned to optimize the categorical cross-entropy loss on our training set by using the Adam optimizer. The learning rate is 5e-3, the dropout rate is 0.3, and the batch size is 4.

1.2.2 Infiltration pattern scoring of PanNETs

The Hover-Net[5] model is used to detect nuclei. It is worth noting that we did not develop another detection/segmentation network specifically for our data but used an already trained model since obtaining nucleus annotations is one of the most resource-intensive and challenging tasks in digital pathology and since the Hover-Net model led to adequately accurate segmentation for the rest of our pipeline. Subsequently, an embedding for each node is calculated by locating a $h \times h$ window centered on the node centroid and extracting features from the encoder of a pre-trained ResNet-34 network[6]. In the experiments, we set $h = 72$ considering the resolution of patches and an average nucleus size in our dataset. The graph topology is then defined by assigning edges between the detected cells, according to the principle that histomorphologically more similar cells in closer proximity exhibit stronger interactions, while the interactions between dissimilar and distant cells are comparatively weaker[4]. To this end, for each graph node n_i, its k most similar nodes $S_i = \{n_1, ..., n_j, ..., n_k\}$ are determined based on the cosine similarity between the feature embeddings

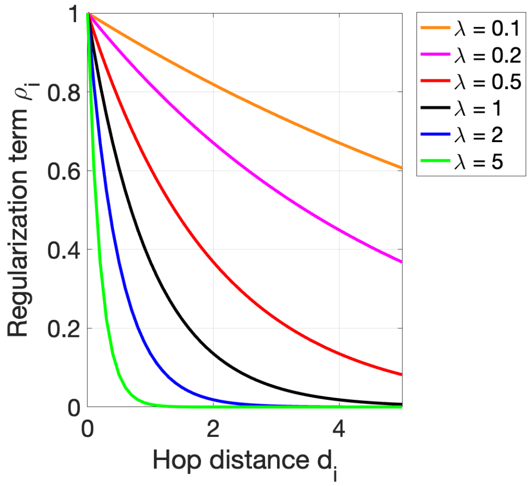

Figure S3: Effects of selecting the decay factor λ to the regularization term ρ_i for the hop distance i.

of the nodes, and an edge is assigned between the nodes n_i and $n_j \in S_i$ if the Euclidean distance between them is less than a threshold τ. By doing so, we aim to model histomorphological and spatial relations of the cells at the same time, which facilitates a more accurate approximation of the inherent connectivity patterns in PanNET cells. In our experiments, we used the HistoCartography framework[7] for cell-graph construction, and set $k = 5$ and $\tau = 75$ pixels considering the cell characteristics in our data.

The GNN is constructed with the architecture containing three graph isomorphism network (GIN) layers[14] enhanced with LSTM jumping knowledge[15]. Additionally, for each node, the features extracted from the ResNet-34 model are used as its embedding, and the sum readout strategy is employed to integrate information from the neighbors of the node. This classifier uses an MLP that has two layers with 128 hidden units as the last layers of the GNN. It uses the Adam optimizer with a learning rate of 0.001 and a batch size of 16.

To introduce *InfiltrationLoss*, let $X_\omega = \{x_i, d_i\}$ be a set of representative tumor-NNP interface patches for a given whole slide image ω, where x_i denotes the i-th patch image and d_i is the hop distance from x_i to the closest patch in the border of the largest TRoI (Figure S2d). Then, for each x_i, the regularization term is defined as $\rho_i = \exp(-\lambda \cdot d_i)$. The decay factor λ determines the amount of this decrease (Figure S3) and selected as 0.5 in our experiments. For the training patch x_i, *InfiltrationLoss*, $L_i = (1 + \rho_i)\text{CE}_i$, is defined by integrating the regularization term ρ_i to the standard categorical cross-entropy loss CE_i.

2 Supplementary Results

To train and test the CNN classifier for PanNET, NNPP, and stroma classification, the data was split into training, validation, and test sets with ratios of 70-10-20 percent, respectively. The training set was used to learn the network weights by backpropagation and the validation set was used for early stopping. Due to randomness in the training of a neural network, runs were repeated three times, and the average F1 scores obtained on the test set were used for evaluation.

For the IPS categorization task, all cases were divided into five-folds, and testing was repeated five times to accurately estimate the classification performance. In each trial, cases in one fold were considered as test instances, whereas cases (and their respective patches) in the remaining four folds were used to train the network. Likewise, due to randomness in the network training, runs were repeated three times for each fold.

Before comparing the reported results with the existing methods, we further analyzed the effectiveness of our selections in the proposed pipeline by performing ablation studies. Here we investigated four factors: graph node embedding, GNN layers, maximum hop distance, and loss function. We changed our selection for each factor, keeping everything else the same, and observed the change in the weighted-F1 score. We also ran five-fold cross-validation and repeated the experiments three times for each fold. This analysis is summarized in Figure S4. The first two factors were directly related to GNN settings. Our pipeline used deep learning-based features extracted from a pre-trained ResNet-34 network as the graph node embedding. We first compared it with randomly generated features to assess the impact of graph topology alone on classification. It led to 63.15 percent weighted-F1 score, emphasizing the use of graph node characteristics in the learning process. Then, we characterized nodes with handcrafted morphological features[16]. A lower weighted-F1 score (71.41 percent) was consistent with the general consensus that learning features directly from data is more effective than defining the features externally. The second factor was related to the GNN architecture. In our pipeline, we used a GNN with graph isomorphism network (GIN) layers[14]. Next, we compared it with two other state-of-the-art GNNs, namely, a graph attention network (GAT)[12] and a graph network with principal neighborhood aggregation (PNA)[3]. Unlike traditional convolution-based GNNs, GATs dynamically employ attention mechanisms to prioritize information from neighboring nodes, making the model adaptive

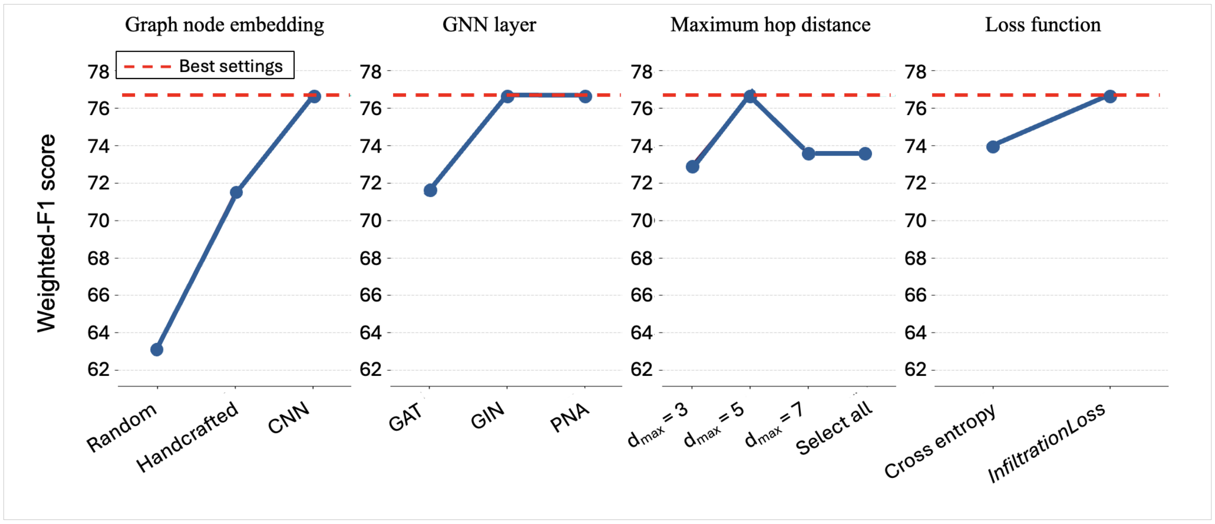

Figure S4: Analysis of the effectiveness of our selections in the proposed pipeline. For four factors, the average weighted-F1 score obtained on the test folds over the 15 runs (five folds and three runs for each fold) as a function of the selection.

to node-specific relational features. GINs improve their capability by utilizing a learnable aggregation function that helps the network distinguish different graph structures. PNAs introduce diverse aggregators and degree-scalers, uniquely capturing local graph features and structural variance. Our experiments showed that using a more advanced aggregation strategy improved the results. The GIN and PNA gave the same weighted-F1 score (76.70 percent) whereas the GAT led to 71.57 percent weighted-F1 score.

The next two factors were related to our proposal to emphasize the tumor-NNP interface when characterizing infiltration patterns and to integrate this emphasis into the GNN training. Our pipeline selected representative PanNET patches that were at most $d_{\text{max}} = 5$ hops away from the edge of the largest TRoI. In our experiments, we observed that a smaller value was not enough to select a sufficient number of patches for accurate characterization, whereas a larger value resulted in losing infiltration characteristics specific to the tumor-NNP interface. In the latter case, the model selected most of the patches in the TRoI and converged to the case where all PanNETs patches were used (as seen in Figure S4, it converged to the case where the entire set of patches was used). Lastly, we investigated the effect of our proposed *InfiltrationLoss* function, which prioritized learning the patterns in patches closer to the tumor-NNP interface. We compared it to the GNN trained with the standard cross entropy loss. The results showed that our proposal improved performance by approximately three percent (from 73.95 to 76.70 percent), highlighting

the importance of integrating domain knowledge into network training.

3 Supplementary Discussion

First, we compared the proposed solution with the pixel-based CNN classifier, Patch-CNN, which was trained on computationally annotated PanNET patches. However, unlike ours, this classifier took patch pixels directly as input and did not use any graphs in its learning process. We also compared the proposed model with three other state-of-the-art GNN-based methods that employed graph neural networks, but their graph construction and learning differed. The Patch-GCN[2] divided a WSI into patches, considered each patch as a node, and constructed a graph by assigning edges between each patch and its adjacent neighbors. The CGC-Net[16] constructed a graph on cell nuclei, similar to ours, but used handcrafted features as node embeddings and GraphSAGE in graph learning. The HACT-Net[10] integrated graphs constructed on both cell nuclei and tissue regions; it used deep learning-based features as node embeddings and PNA layers in the GNN architecture. Table 2 of the main manuscript provides a comparative evaluation of the performance of these methods. In this comparison, all GNN-based methods significantly outperformed the Patch-CNN. Moreover, that Patch-GCN was the baseline among the GNN-based classifiers. Consistent with our previous discussion, these two observations indicate the effectiveness of representing patches as graphs constructed on their cell nuclei. Both the HACT-Net and the proposed *InfiltrationLoss*-GNN improved the F1-scores of the CGC-Net, especially for the IPS2 and IPS3 classes. This might be attributed to the advantage of using deep learning-based node embeddings over handcrafted features and the superiority of GIN[14] and PNA[3] layers. At that point, it is also worth comparing the HACT-Net and the variation of our model that was trained on the selected representative patches but without optimizing the *InfiltrationLoss* function (see the result corresponding to cross-entropy in Figure S4). The HACT-Net method took advantage of hierarchically integrating cell and tissue graphs, while our variation utilized concentration on only the selected patches that better represented the infiltration patterns. Although they had very similar scores (73.90 and 73.95 percent weighted-F1 scores, respectively), the latter offered a more lightweight deep learning model.

Lastly, we compared our results with a multiple instance learning algorithm commonly used in histopathology, which is called CLAM[8]. Its results are also reported in Table 2 of the main

manuscript. All F1-scores obtained by CLAM were lower than those obtained by the proposed *InfiltrationLoss*-GNN model. Additionally, although it gave a better weighted F1-score (62.33 percent) compared to the CNN classifier, this performance was lower than those of all graph-based classifiers reported in this table. This lower performance might be attributed to the following: while the CLAM algorithm was effective in identifying tumor and non-tumor regions in general, it may struggle to capture finer details with its features required to effectively categorize PanNET infiltration patterns, for which nuanced morphological differences are important. Additionally, the CLAM algorithm must identify the most informative patches on its own, without using any external knowledge, which might be important for a given application. On the other hand, the ability of our proposed model to make use of pathology domain knowledge, both in selecting representative patches and in training a model through a custom loss function, was important to make more accurate and robust predictions for PanNET categorization. These are the main strengths of the proposed model.

References

1. Bankhead P FJeaLoughrey MB (2017) Qupath: Open source software for digital pathology image analysis. Sci Rep 7(1):1–7. https://doi.org/https://doi.org/10.1038/s41598-017-17204-5

2. Chen RJ SMeaLu MY (2021) Whole slide images are 2d point clouds: Context-aware survival prediction using patch-based graph convolutional networks. In: Med Image Comput Comput Assist Interv, pp 339–349, https://doi.org/https://doi.org/10.1007/978-3-030-87234-2_32

3. Corso G BDeaCavalleri L (2020) Principal neighbourhood aggregation for graph nets. Adv Neural Inf Process Syst 33:13260–13271. https://doi.org/https://doi.org/10.48550/arXiv.2004.05718

4. Francis K PB (1997) Effective intercellular communication distances are determined by the relative time constants for cyto/chemokine secretion and diffusion. Proc Natl Acad Sci U S A 94(23):12258–12262. https://doi.org/https://doi.org/10.1073/pnas.94.23.12258

5. Graham S RSeaVu QD (2019) Hover-net: Simultaneous segmentation and classification of nuclei in multi-tissue histology images. Med Image Anal 58:101563. https://doi.org/https://doi.org/10.1016/j.media.2019.101563

6. He K RSSJZhang X (2016) Deep residual learning for image recognition. In: IEEE Conf Comput Vis Pattern Recognit, pp 770–778, https://doi.org/https://doi.org/10.1109/CVPR.2016.90

7. Jaume G AVeaPati P (2021) Histocartography: A toolkit for graph analytics in digital pathology. In: MICCAI Workshop Comput Pathol, pp 117–128, https://doi.org/https://doi.org/10.1007/978-3-030-87237-3_10

8. Lu MY CTeaWilliamson DFK (2021) Data-efficient and weakly supervised computational pathology on whole-slide images. Nat Biomed Eng 5(6):555–570. https://doi.org/https://doi.org/10.1038/s41551-020-00682-7

9. Macenko M MJeaNiethammer M (2009) A method for normalizing histology slides for quantitative analysis. In: IEEE Int Symp Biomed Imaging, pp 1107–1110, https://doi.org/https://doi.org/10.1109/ISBI.2009.5193250

10. Pati P FRAeaJaume G (2022) Hierarchical graph representations in digital pathology. Med Image Anal 75:102264. https://doi.org/https://doi.org/10.1016/j.media.2021.102264

11. Tellez D BPeaLitjens G (2019) Quantifying the effects of data augmentation and stain color normalization in convolutional neural networks for computational pathology. Med Image Anal 58:101544. https://doi.org/https://doi.org/10.1016/j.media.2019.101544

12. Veličković P CAeaCucurull G (2017) Graph attention networks. arXiv 1710.10903. https://doi.org/https://doi.org/10.48550/arXiv.1710.10903

13. Veta M VDPVMPluim JP (2014) Breast cancer histopathology image analysis: A review. IEEE Trans Biomed Eng 61(5):1400–1411. https://doi.org/https://doi.org/10.1109/TBME.2014.2303852

14. Xu K LJJSHu W (2018) How powerful are graph neural networks? arXiv 1810.00826. https://doi.org/https://doi.org/10.48550/arXiv.1810.00826

15. Xu K TYeaLi C (2018) Representation learning on graphs with jumping knowledge networks. In: Int Conf Mach Learn, pp 5453–5462, https://doi.org/https://doi.org/10.48550/arXiv.1806.03536

16. Zhou Y AKNeaGraham S (2019) Cgc-net: Cell graph convolutional network for grading of colorectal cancer histology images. In: IEEE/CVF Int Conf Comput Vis Work, https://doi.org/https://doi.org/10.48550/arXiv.1909.09548
